# Editorial: Model organisms in predictive toxicology 2023

**DOI:** 10.3389/fphar.2025.1609126

**Published:** 2025-04-17

**Authors:** Cristina Luceri, Abdulahad Dogan, Angel León-Buitimea

**Affiliations:** ^1^ Department of Neurosciences, Psychology, Drug Research and Child Health, University of Florence, Florence, Italy; ^2^ Faculty of Pharmacy, Van Yuzuncu Yil University, Van, Türkiye; ^3^ Tecnologico de Monterrey, School of Medicine and Health Sciences, Monterrey, Mexico

**Keywords:** *In silico* models, chicken embryos, zebrafish, rats, 3D model brainspheres

The identification of adverse effects induced by a test compound is generally based on the collection and analysis of a variety of toxicological studies that include integrated methodological approaches (Schmeisser et al., 2023) ranging from in-chemico (measurement of chemical reactivity), *in silico* (predictive measures based on computational models), *in vitro* (cell cultures, subcellular fractions, organoids), *in vivo* (rodents or other species) and human observations (scientific, epidemiological and clinical data), called *lines of evidence* (Johnson et al., 2022; Browne et al., 2024).

Model organisms are an invaluable resource for both basic and applied research, enabling studies to predict, model, and identify mechanisms of action. This Research Topic comprises four articles that investigate the toxicological effects of compounds with biological activity using *in silico* models, chicken embryos, zebrafish, rats, and 3D model BrainSpheres.

Khosravi et al. investigated the effects of amphotericin B (Amp B) and its liposomal formulation, AmBisome, on fetal organs using *in silico* and *in vivo* assays in chicken embryos. Amp B is a second-line treatment for leishmaniasis, a significant disease in tropical and subtropical regions, with reported side effects affecting various organs. In chicken embryos, the authors studied pathological changes, alterations in angiogenesis, and apoptosis induced by Amp B and AmBisome, using molecular docking. Using molecular docking, they predicted the affinity of Amp B and AmBisome to proteins associated with angiogenesis and apoptosis processes. The ADME toxicity prediction indicates that AmBisome exhibits a superior pharmacological effect compared to AmB, suggesting its potential advantages as a treatment strategy for leishmaniosis during pregnancy over AmB.

The original research study by Chen et al. examines the effects of cysteamine (β-mercaptoethylamine) on skeletal development and motor behavior in zebrafish embryos, as well as the involvement of the Notch signaling pathway. This feed additive in livestock and aquaculture is also used as a targeted agent for the treatment of cystinosis. A small proportion of patients have experienced side effects affecting bones, skin, blood vessels, nerves, and muscles. Moreover, reports on experimental models have indicated its potential harm, particularly skeletal toxicity with an unclear mechanism. Exposure to various concentrations of cysteamine resulted in significant and dose-dependent mortality, skeletal deformities, reduced mobility, increased oxidative stress, and altered gene expression related to key cellular processes. The study also shows that disruption of Notch signaling may underlie these adverse effects, as co-administration of sodium valproate partially ameliorates the cysteamine-induced downregulation of Notch signaling.

Another study in the Research Topic, also by Ye et al. assesses subacute toxicity in rats. The 28-day oral toxicity study in rodents (OECD Test Guideline 407) is a protocol designed to assess the toxicity of chemicals through daily oral administration to rodents over a limited period, with the intent of examining effects on a wide variety of potential toxicity targets. During the administration period, the animals are closely observed daily for signs of toxicity. At the end of the test period, it is possible to fully explore the short-term effects of the test compound, its toxicokinetics, any noticeable histopathological effects, and the eventual reversal after recovery. Here, these authors tested the subacute toxicity of a μ-opioid receptor agonist with potent antinociceptive activity and reduced respiratory suppression, administered at various doses via intravenous infusion over 28 days. They were able to evaluate clinical and biochemical observations, gross necroscopy, histopathologic evaluations, and the measurement of toxicokinetic parameters. Although the OECD Guidelines for the Testing of Chemicals are periodically revised considering scientific advances, regulatory agencies still require *in vivo* animal studies to assess drug safety prior to clinical trials. Otherwise, bringing toxicological screening into the 21st century will require the more extensive use of complex cellular models, induced pluripotent stem cells (iPSCs), organoids, and tissues-on-a-chip, as well as data analysis tools, including machine learning applications.

Nunes et al. utilized a 3D model derived from human iPSCs, known as BrainSpheres, to evaluate the neurotoxicity of amiodarone, a lipophilic antiarrhythmic medication. This model comprises electrophysiologically active neurons, astrocytes, and oligodendrocytes, which are capable of forming compact myelin sheaths around axons. Using these BrainSpheres, they developed a model of compartmental distribution kinetics. They observed a time- and concentration-dependent increase in the neurotoxic effects of amiodarone, attributed to cellular accumulation of the drug following repeated administration. These results suggest that human cells are more sensitive to amiodarone than rodent cells, highlighting the importance of innovative *in vitro* systems in better predicting chemical safety and neurotoxic effects in humans.

We hope this Research Topic provides valuable insights into the model organisms that are significant to the field of Predictive Toxicology as a whole.
